# Prostate Cancer Presenting as Hip Pain at the Chiropractic Office: A Case Report and Literature Review

**DOI:** 10.7759/cureus.34049

**Published:** 2023-01-21

**Authors:** Eric Chun-Pu Chu, Wai Ting Lee

**Affiliations:** 1 Department of Chiropractic and Physiotherapy, New York Chiropractic and Physiotherapy Centre, Hong Kong, HKG

**Keywords:** prostate cancer, hip metastasis, hip pain, hip cancer, chiropractic

## Abstract

Prostate cancer is one of the most common cancers found in males, and it tends to metastasize to bony parts such as the hip, spine, and pelvis, resulting in pain and/or radicular pain, which can present similarly to musculoskeletal complaints. The lack of routine screening and musculoskeletal symptoms present challenges in the diagnosis of prostate cancer.

We report the case of a 62-year-old male with no history of cancer and no previous prostate cancer screening who visited a chiropractor for the care of worsening left hip pain after a marathon. The patient visited other healthcare providers and was suggested to have degenerative conditions; he received nonsteroidal anti-inflammatory medication, physiotherapy, and acupuncture. Given the patient’s limited improvement by other providers and neurological symptoms, the chiropractor requested lumbar spine radiography, which revealed suspected bone metastasis, and ordered a hip MRI accordingly. MRI findings suggested prostate cancer, and the chiropractor referred the patient to an oncologist, who performed additional imaging and testing to make a presumptive prostate cancer diagnosis. A literature search found nine cases of undiagnosed prostate cancer presenting to a chiropractor for care. All patients included in this case were older males with no previous prostate screening or bone metastasis.

The study is focused on the need for a comprehensive evaluation of patients with hip pain during a chiropractic visit due to the chances of prostate cancer. There are higher chances of ignoring cancer symptoms during a hip examination. Comprehensive evaluation and advanced imaging could help chiropractors detect patients with prostate cancer.

## Introduction

Hip pain is a common and incapacitating symptom among adults aged 60 and older [1]. Infection, aortoiliac insufficiency, and bone metastasis are rare causes of hip pain [1]. Prostate cancer incidence and mortality also correlate with old age [2]. Prostate cancer is the leading type of cancer resulting from bone metastasis and often invades the spine, pelvis, hips, or ribs [3]. In later stages, patients suffer from metastasis to the bones and typically present with severe bone pain, nerve root pain, neurological deficits, or bladder dysfunction. In advanced stages of metastasis, it can travel to the lungs, liver, pleura, and adrenal glands [4]. Although it is uncommon, bone metastasis can lead to chronic and disabling hip pain [1].

Prostate-specific antigen (PSA) testing is used for prostate cancer screening. However, testing remains debated as there is no significant evidence to improve the prostate cancer mortality rate, which leads to different PSA testing recommendations globally [5,6]. Researchers suggested that PSA testing is frequently used in urological settings, as urologists are more convinced about testing than general practitioners [7]. A survey suggested that only 5% of the male respondents conducted PSA testing for prostate cancer screening in Hong Kong [8], as in this case. One study suggested that limited prostate cancer screening and prostate cancer characteristics caused patients to remain undiagnosed until later stages of the disease [4,9].

Chiropractors are primary healthcare providers who see patients with neuromusculoskeletal complaints [10]. It is not common for chiropractors to encounter severe pathologies such as cancer in their practice [11]. A study in Hong Kong revealed that chiropractors only encounter malignancy in approximately 0.25% of adults with low back pain [12]. Regarding prostate cancer presentation, it is important for chiropractors to identify and refer such patients to appropriate healthcare professionals for further investigation or treatment, due to the impact on patients if left undiagnosed or untreated.

Considering increased prostate cancer incidence and the potential for chiropractors to encounter undiagnosed cancer in the elderly population, we present a rare case of left hip pain that was secondary to bone metastasis from prostate cancer, leading to severe refractory hip pain and serious functional and quality of life impairments. We highlight the role of chiropractors in identifying bone metastasis and the necessity of communicating clinical findings to radiologists and oncologists for further investigation and co-management.

## Case presentation

Hip pain is a common and incapacitating symptom among adults aged 60 and older [1]. Infection, aortoiliac insufficiency, and bone metastasis are rare causes of hip pain [1]. Prostate cancer incidence and mortality also correlate with old age [2]. Prostate cancer is the leading type of cancer resulting from bone metastasis and often invades the spine, pelvis, hips, or ribs [3]. In later stages, patients suffer from metastasis to the bones and typically present with severe bone pain, nerve root pain, neurological deficits, or bladder dysfunction. In advanced stages of metastasis, it can travel to the lungs, liver, pleura, and adrenal glands [4]. Although it is uncommon, bone metastasis can lead to chronic and disabling hip pain [1].

Prostate-specific antigen (PSA) testing is used for prostate cancer screening. However, testing remains debated as there is no significant evidence to improve the prostate cancer mortality rate, which leads to different PSA testing recommendations globally [5,6]. Researchers suggested that PSA testing is frequently used in urological settings, as urologists are more convinced about testing than general practitioners [7]. A survey suggested that only 5% of the male respondents conducted PSA testing for prostate cancer screening in Hong Kong [8], as in this case. One study suggested that limited prostate cancer screening and prostate cancer characteristics caused patients to remain undiagnosed until later stages of the disease [4,9].

Chiropractors are primary healthcare providers who see patients with neuromusculoskeletal complaints [10]. It is not common for chiropractors to encounter severe pathologies such as cancer in their practice [11]. A study in Hong Kong revealed that chiropractors only encounter malignancy in approximately 0.25% of adults with low back pain [12]. Regarding prostate cancer presentation, it is important for chiropractors to identify and refer such patients to appropriate healthcare professionals for further investigation or treatment, due to the impact on patients if left undiagnosed or untreated.

A 62-year-old male construction worker with a history of hypertension and hyperlipidemia presented with a seven-day history of deep and aching pain in the front of the left groin region, radiating down the thigh to the knee. The patient’s pain intensity was reported as four out of 10 on the numeric pain rating scale after a marathon. Although symptoms were alleviated by rest, the insidious pattern of pain and stair climbing primarily affected his daily activities and work at the construction sites. He denied having any trauma, a family history of similar illnesses, unexplained weight loss, fever or night sweats for unknown reasons, fatigue, or changes in bowel habits or bladder function. He had no family history of metastatic diseases or hip disorders. The patient was a marathoner, and he reported that the symptoms had negatively impacted his running. His World Health Organization Quality of Life Score (WHOQOL) was recorded at 50%.

The patient was a 70 kg, 170 cm Asian male who was taking medication for hypertension and hyperlipidemia at the time and initially consulted his primary care physician for hip pain after running a marathon. He was diagnosed with a soft tissue injury of the hip joint and treated with nonsteroidal anti-inflammatory medication and sports rehabilitation without further investigation. No prostate-specific antigen (PSA) testing was recommended for the patient. The sports rehabilitation included hip-strengthening exercises and thermal ultrasound therapy. He also tried a traditional medical practitioner with acupuncture, but none of the therapies provided permanent relief from symptoms. The patient sought chiropractic therapy for conservative management of his hip pain.

**Figure 1:**
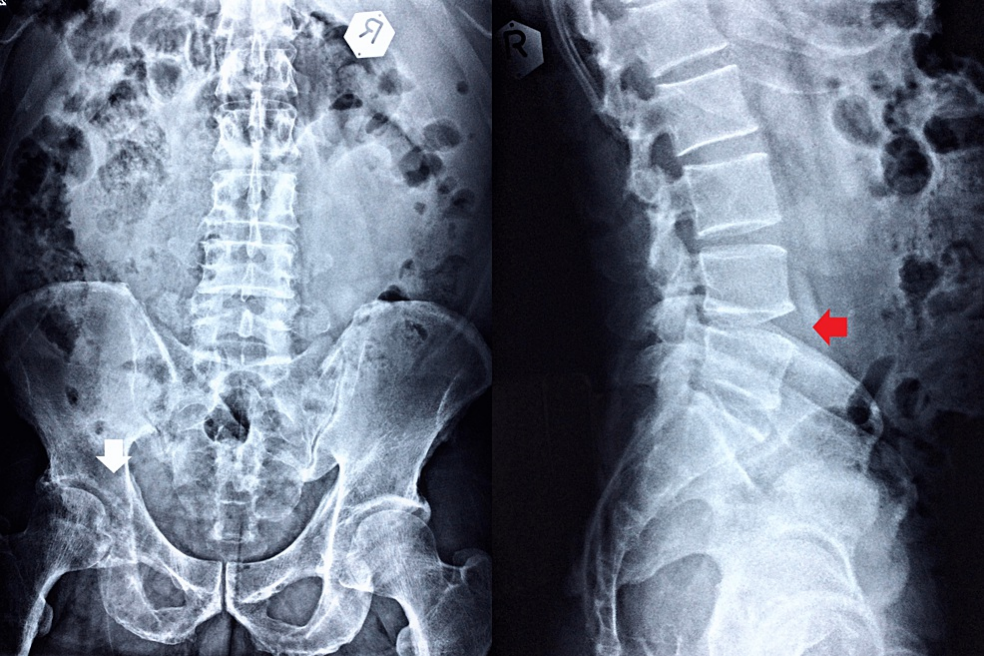
Lumbar and pelvic radiography The anteroposterior and lateral views appear to have reduced L4-5 disc space and a marginal anterior osteophyte in the lumbosacral spine (LS) vertebral body (red arrow). Multiple lucent foci are also found on the left pelvic rim, femoral head, and proximal body (white arrow).

**Figure 2:**
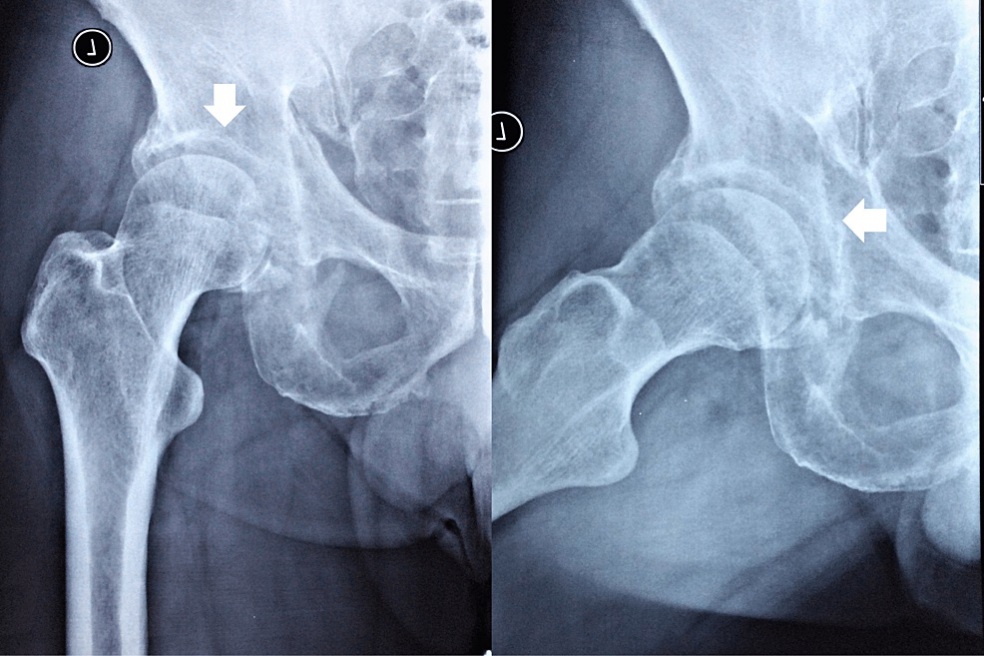
Hip radiography Hip radiographs identified multiple lucent foci on the left pelvic rim, femoral head, and proximal body (white arrows).

Upon examination by the chiropractor, the patient walked normally with a steady gait pattern in terms of balance, strength, and coordination. He had a normal range of motion in the lumbar spine, but the overall range of motion of the left hip was limited due to pain. Both flexion abduction external rotation (FABER) of the hip and sacroiliac joint spring tests reproduced pain in the left hip, lumbosacral spine, and sacroiliac joint. Orthopedic examinations of the knees and lumbar spine were negative. Motor strength testing of the lower extremity was graded 4/5 in left hip flexion (on the Medical Research Council Scale). Palpation identified hypertonicity at the paraspinal muscles, gluteal muscles, iliotibial band, lateral rotators, and adductors of the left hip. The differential diagnosis included lumbar disc radiculopathy, pelvic disorders, and hip pathology. The chiropractor took lumbar, pelvic (Figure 1), and hip (Figure 2) radiographs as an initial imaging test and identified multiple lucent foci on the left pelvic rim, femoral head, and proximal body. The chiropractor then prescribed a second hip MRI for a definite diagnosis and started a trial of care to reduce his symptoms.

**Figure 3:**
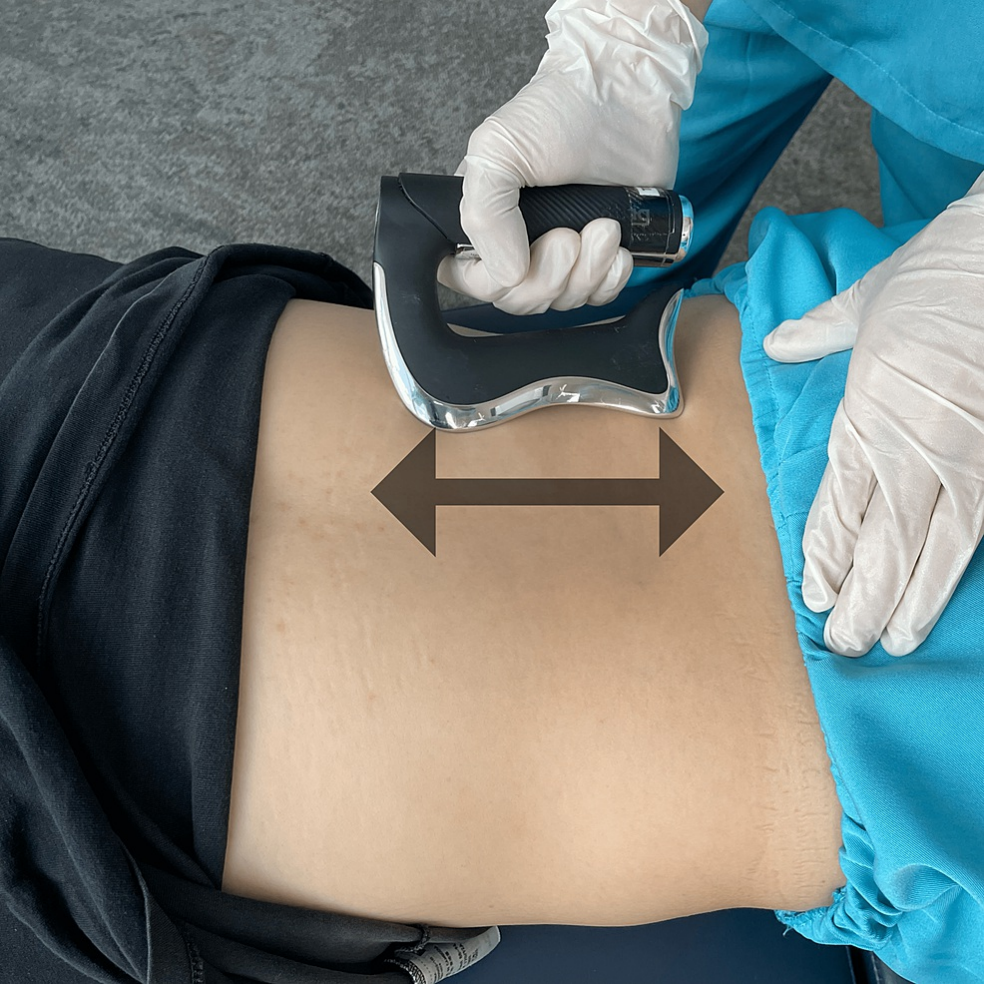
Muscle pain relief Utilization of an instrument-assisted soft tissue mobilization device (Strig Pro, STRIG, Korea) stroking the skin surface of the lumbar paraspinal muscles, gluteal muscles, iliotibial band, lateral rotators, and adductors of the left hip repeatedly with the use of lubricating cream around the left hip joint.

The chiropractor started a trial treatment with gentle manual therapy and strengthening exercises while waiting for the MRI examination results. By tilting the equipment's gravity to act on the trunk, the patient remained seated as the equipment was slowly tilted through different angles of rotation and inclination for 20 minutes per treatment to strengthen the core muscles. The patient had to stabilize a hip-width-sized fit ball between the legs to target hip muscle endurance (AllCore360° Core Training System®, AllCore360°, GA, USA). The chiropractor also reduced muscle tightness by using an instrument-assisted soft tissue mobilization device (Strig Pro, STRIG, Korea) and stroking the skin surface of the hypertonic muscles repeatedly with lubricating cream (Figure 3). The treatments were applied three times a week for one week. The patient reported relief from hip pain, which subsided to moderate severity over the first week.

**Figure 4:**
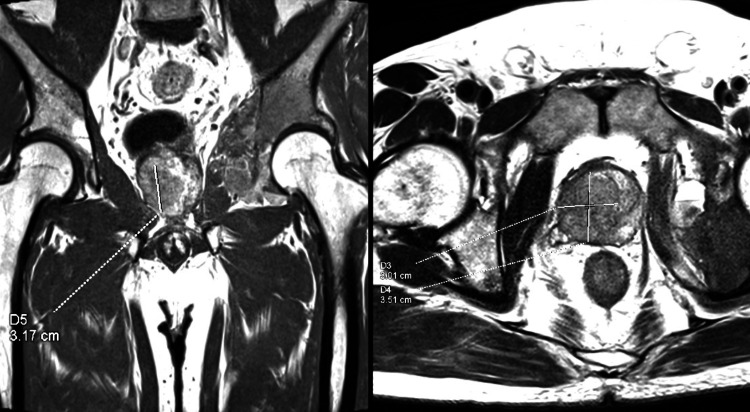
MRI of the left hip Hip MRI identified a T2W hypointense mass (3.01x3.51x3.17cm) with marked restricted diffusion that is noted at the prostate from base to apex, with the epicenter on the right side and extension to the left side, highly suggestive of prostate metastasis.

The MRI was analyzed by a radiologist, who reported suspicion of prostate metastasis (Figure 4).

**Figure 5:**
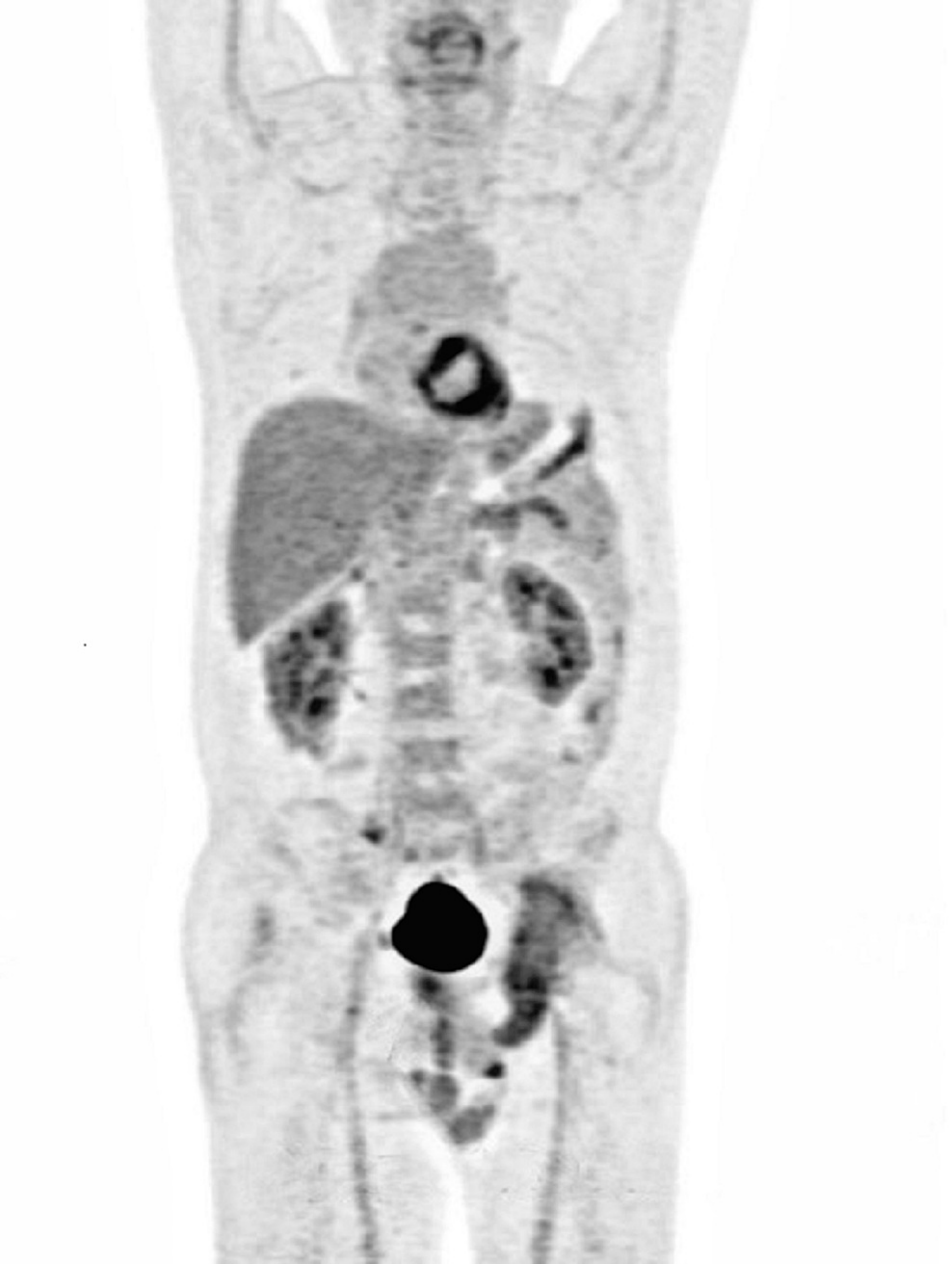
F18 FDG whole-body PET-CT Radiological carcinoma of the prostate with bone and lung metastases.

**Figure 6:**
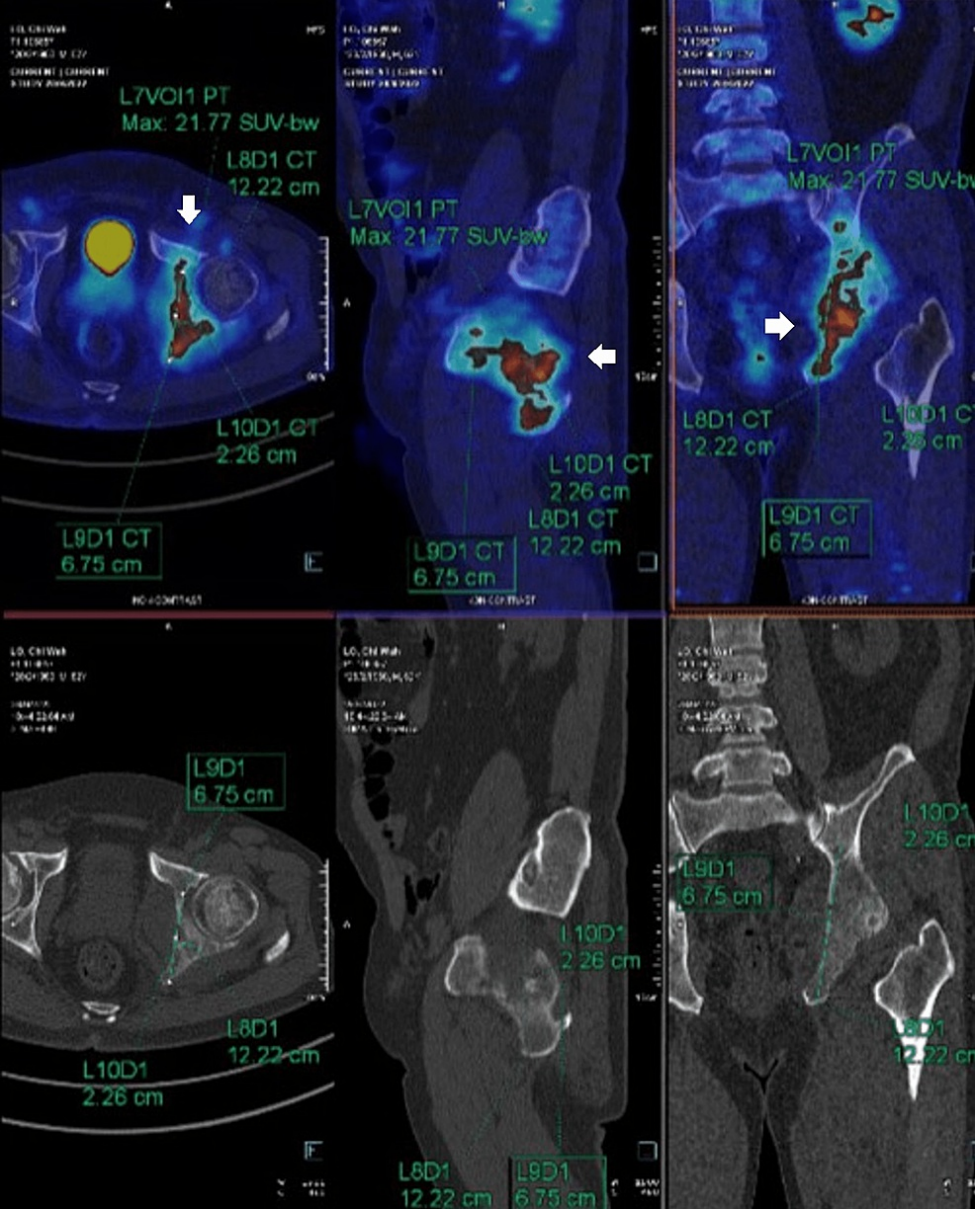
F18-PSMA whole-body PET and CT The images confirmed the carcinoma of the prostate with numerous lung and bone metastases (white arrows). More bone metastases are detected on PSMA examination. Spotty right external iliac lymph nodes, as described, represent early lymph node metastasis.

The chiropractor then consulted a clinical oncologist at the same institute and scheduled the patient for a visit the following day. The clinical oncologist also examined the patient the following day and ordered an F-fluorodeoxyglucose positron emission tomography (FDG-PET) scan, a prostate-specific membrane antigen positron emission tomography (PSMA-PET) scan, and a biopsy. The PET-CT identified mixed lytic sclerotic lesions involving the medial wall of the left acetabulum and left ischium. Cortical destruction and soft tissue penetration were also observed. This measured about 2.26 x 6.75 x 12.22 cm (SUV max: 21.77). These findings confirmed the presence of bone metastases. A hypermetabolic prostatic lesion was observed involving the right half of the prostate, measuring 2.37 x 2.81 x 3.86 cm (SUV max: 5.71), compatible with prostate carcinoma. Numerous subcentimeter nodules were scattered in both lungs, suggestive of lung and lymph node metastasis (Figures 5, 6).

The oncologist suggested a combination of treatments, including androgen deprivation, novel hormonal agents, systemic chemotherapy, and bone resorptive agents. The patient was prescribed subcutaneous Enantone (11.25 mg) and subcutaneous denosumab (120 mg) at the clinic and deprivation and bone resorption agents at the public hospital.

In the interim, the oncologist cleared the patient to continue receiving chiropractic therapies, as they initially provided relief. The oncologist and chiropractor decided to continue using rehabilitation exercises and gentle manual joint mobilization for 10 sessions. Additionally, hip rehabilitation home exercises were advised to be practiced daily for 15 minutes. As a precaution against the risk of pathological fracture and metastasis, which is considered a contraindication for spinal manipulation, only gentle therapy was provided due to the presence of known hip metastases.

Four weeks later, the prostate biopsy returned from the public hospital, and the adenocarcinoma was confirmed. The patient’s hip range of motion had already improved by 50%, and his WHOQOL score improved from 50% to 70%. The patient noted that he resumed most of his usual daily activities and work; however, he avoided running marathons. He only walked daily during grocery shopping and occasionally dined out at family gatherings. He was instructed to perform home-based rehabilitation activities while receiving medical treatment. The patient died from a lung infection seven months after being diagnosed with prostate cancer.

## Discussion

This case illustrates an elderly male athlete who previously visited other healthcare providers and was treated for a degenerative condition. The chiropractor reviewed the patient's condition and ordered a hip radiography. Red flags were recognized, and an MRI was ordered immediately, which was suggestive of prostate cancer. The patient was then referred to the clinical oncologist for further investigation and subsequent treatment.

This case demonstrates the difficulty of identifying prostate cancer in a patient presenting with what seemed to be a musculoskeletal disorder. Primary healthcare providers, such as chiropractors, should be aware that this condition can present with various symptoms to mask the underlying cause [3,4]. In the current case, the lack of PSA testing could be one of the factors contributing to the delayed diagnosis. Although the effectiveness of PSA testing remains debated [5], a comprehensive examination that includes a physical examination, radiography imaging, and medical laboratory testing should be considered with respect to patient history [8]. Providers should acknowledge that radiography alone might not be sufficient for detecting malignancy, and advanced imaging techniques such as MRI or fluoro-2-deoxy-d-glucose positron emission tomography (FDG-PET) should be ordered for suspected malignancy or metastasis [13].

The chiropractor was responsible for patient diagnosis and referral to a suitable healthcare provider. However, in this case, the chiropractor was able to provide symptomatic relief to the patient. The current conservative management of pain symptoms associated with prostate cancer is based on the careful management of traditional musculoskeletal pain. There is no research and no standard protocol for chiropractic rehabilitation in the management of patients with cancer. The conservative management of oncology research is rarely studied, and our case is an additional reference for future clinicians. As most chiropractors would not have oncology cases within their scope of practice, there is insufficient evidence or protocols regarding chiropractic care for patients with cancer [14,15]. The chiropractic spinal manipulation technique is contraindicated in neoplastic diseases [16]. Nonetheless, with careful consideration, certain low-force techniques or exercises could be beneficial and appropriate for patients with these conditions [15].

A literature search was conducted on December 28, 2022, using the search terms "chiropractic," "chiropractor," "prostate," and "prostatic" in PubMed, Google Scholar, and the Index to Chiropractic Literature. All searches were limited to those studies written in English. Search results revealed nine published cases of undiagnosed prostate cancer from 1986 to 2022 that sought chiropractic care, which led to suggested or confirmed prostate cancer [17-24]. Spinal region pain (cervical, thoracic, and lumbar) was the most common complaint in 7/10 (70%) of these cases, including the current case. Only two cases reported elevated PSA levels in the initial investigation [18,22].

As with previously published cases, the patients, in this case, were all older males with no history of PSA screening who were later diagnosed with prostate cancer that had metastasized to other body parts. While the patient initially complained of hip pain and no back pain, this difference might have contributed to the delayed diagnosis, which commonly presents with low back pain if metastasis tends to involve the thoracolumbar and sacral regions [4,25]. This case serves as a reminder that providers should be aware that prostate cancer can produce various symptoms and should be considered a differential diagnosis in older males.

Aside from case reports and survey studies, the incidence rate of undiagnosed cancer presenting to the chiropractor remains unknown [12,17,24]. Given the possible complication caused by cancer metastasis, if not previously identified, it is essential for chiropractors to be aware of such patients.

This case was confirmed using detailed clinical and advanced imaging findings. The involvement of the radiology and oncology teams reinforced this case. Additionally, this case examined other published cases and revealed similarities and differences between these cases. Similar to previously studied cases, ours involved an elderly man who had never undergone PSA screening and was confirmed to have prostate cancer metastases. However, the present case is unique because he presented with hip pain as his chief complaint. In addition, the current case is one of the three reports in which an MRI was performed earlier in the evaluation process. Although this difference may be due to the fact that previous examples were reported some years ago when MRI was not widely utilized, the present case also demonstrates the value of early MRI for chiropractors to detect spinal metastases. However, the current case may not be broadly generalizable. As the scope of practice varies among countries, chiropractors might not have the authority to request advanced imaging, such as an MRI. This case might not be presented to the chiropractor if the setting routinely conducts prostate cancer screening for citizens. Furthermore, this case lacked biopsy findings as the examination was not conducted by the same healthcare organization and the report could not be retrieved. And finally, a true case of death was not identified. 

## Conclusions

Patients with undiagnosed prostate cancer might visit a chiropractor for care if the cancer metastasizes to other body parts, causing various symptoms such as hip pain and/or radicular pain. Chiropractors should be aware of red flags presented on plain imaging; if any, they should request advanced imaging appropriately, which might aid in the timely diagnosis of prostate cancer. Depending on the country’s guidelines, chiropractors should perform a comprehensive examination when encountering an undiagnosed cancer patient. If the clinical or imaging examination suggests cancer, it is important for the chiropractor to refer patients to suitable healthcare providers, such as oncologists, to ensure timely treatment.
